# Supplementary material for: When to use commuting zones? An empirical description of spatial autocorrelation in U.S. counties versus commuting zones
Source: PLoS One. 2022 Jul 13;17(7):e0270303. doi: 10.1371/journal.pone.0270303 (PMC9278745; doi:10.1371/journal.pone.0270303)
Supplement: S1 Table — Significance levels: ***<1%, **<5%, *<10% Notes: Table provides the descriptive statistics for both counties and the CZs values. The numbers by the industries represent their two-digit NAICS code classification. Abbreviations: LQs, Location Quotients; NAICS, North American Industrial Classification System; Voters in 2000, percent of eligible population that vote in presidential election (2000) per 1000 population; Total bank deposits, commercial banks and savings institutions—total deposits (thousands); std. dev., standard deviation. (DOCX) [file pone.0270303.s001.docx]

| **S1 Table. Additional Descriptive Statistics in Counties and CZs** | | | | | |
| --- | --- | --- | --- | --- | --- |
| Ecological domain | Measure/variable | County mean | County std. dev. | CZ  mean | CZ  std. dev. |
| Entrepreneurial | % workforce self-employed | 9.55 | 5.16 | 9.36 | 4.65 |
|  | Businesses with 1-4 employees | 57.46 | 6.80 | 55.88 | 5.06 |
|  | % creative class | 17.24 | 6.01 | 18.42 | 4.88 |
| Economic | Total bank deposits | 11,971.62 | 78,899.70 | 12,746.03 | 5,215.82 |
|  | % population below poverty | 14.18 | 6.55 | 14.44 | 5.45 |
|  | Unemployment rate | 9.46 | 0.23 | 9.44 | 0.20 |
|  | Per capita income | 17,488.31 | 3,932.29 | 17,612.90 | 3,226.91 |
| Social | Associations per 10,000 | 1.41 | 0.66 | 1.45 | 0.57 |
|  | Third places per 10,000 | 0.78 | 0.50 | 0.81 | 0.41 |
|  | Voters in 2000 per 1000 | 535.66 | 101.86 | 1.91 | 2.04 |
|  | Adherents to civic denominations per 1000 | 298.02 | 178.74 | 300.86 | 158.37 |
| Demographic | % population identify as Black | 8.75 | 14.48 | 8.02 | 12.19 |
|  | % population identify as Hispanic | 6.21 | 12.04 | 7.49 | 12.78 |
|  | % adult population with $\geq$ bachelor’s | 16.51 | 7.80 | 18.09 | 6.24 |
|  | % population age 25 and younger | 34.36 | 4.45 | 35.01 | 4.06 |
|  | % population age 65 and older | 14.81 | 4.11 | 14.68 | 3.60 |
| Industrial LQs | 11: Ag, Forestry, etc. | 4.95 | 12.24 | 3.35 | 6.44 |
|  | 21: Mining, Quarrying, Oil, etc. | 4.76 | 15.02 | 4.06 | 9.93 |
|  | 22: Utilities | 1.99 | 4.44 | 1.61 | 1.64 |
|  | 23: Construction | 1.05 | 0.71 | 0.99 | 0.43 |
|  | 31-33: Manufacturing | 1.36 | 0.99 | 1.21 | 0.73 |
|  | 42: Wholesale Trade | 0.86 | 0.70 | 0.86 | 0.51 |
|  | 44-45: Retail Trade | 1.25 | 0.38 | 1.24 | 0.26 |
|  | 48-49: Transportation and Warehousing | 0.91 | 1.13 | 0.85 | 0.54 |
|  | 51: Information | 0.57 | 0.61 | 0.68 | 0.36 |
|  | 52: Finance and Insurance | 0.75 | 0.52 | 0.77 | 0.34 |
|  | 53: Real Estate and Rental and Leasing | 0.63 | 0.69 | 0.73 | 0.47 |
|  | 54: Prof., Scientific, and Tech. Services | 0.47 | 0.43 | 0.53 | 0.30 |
|  | 55: Management of Enterprises | 0.31 | 0.64 | 0.41 | 0.53 |
|  | 56: Admin., Support, Waste Management, etc. | 0.47 | 0.56 | 0.57 | 0.46 |
|  | 61: Educational Services | 0.69 | 1.46 | 0.76 | 1.05 |
|  | 62: Health Care and Social Assistance | 1.18 | 0.60 | 1.22 | 0.36 |
|  | 71: Arts, Entertainment, and Recreation | 0.93 | 2.09 | 0.95 | 1.17 |
|  | 72: Accommodation and Food Services | 1.10 | 0.67 | 1.17 | 0.47 |
|  | 81: Other Services | 0.98 | 0.41 | 1.00 | 0.27 |
| Significance levels: ***<1%, **<5%, *<10%  *Notes*: Table provides the descriptive statistics for both counties and the CZs values. The numbers by the industries represent their two-digit NAICS code classification.  *Abbreviations*: LQs, Location Quotients; NAICS, North American Industrial Classification System; Voters in 2000, percent of eligible population that vote in presidential election (2000) per 1000 population; Total bank deposits, commercial banks and savings institutions – total deposits (thousands); std. dev., standard deviation. | | | | | |
