# Supplementary material for: When to use commuting zones? An empirical description of spatial autocorrelation in U.S. counties versus commuting zones
Source: PLoS One. 2022 Jul 13;17(7):e0270303. doi: 10.1371/journal.pone.0270303 (PMC9278745; doi:10.1371/journal.pone.0270303)
Supplement: S2 Table — Significance levels: ***<1%, **<5%, *<10% Notes: Table summarizes the Global Moran’s I Test for Spatial Dependence. This table uses an inverse-distance based spatial weighting matrix to produce the Moran’s I Index (displayed), Expected Index, Variance (displayed), z-score (displayed), and p-value (displayed). The weight was constructed in GeoDa 1.16, and test performed with the SPATGSA [62] command in Stata 17/SE. Abbreviations: Voters in 2000, percent of eligible population that vote in presidential election (2000) per 1000 population; Total bank deposits, commercial banks and savings institutions—total deposits; std. err., standard error. (DOCX) [file pone.0270303.s002.docx]

**S2.1 Table. Global Moran’s I for Entrepreneurial, Economic, Social, and Demographic Domains (Counties versus CZs), Inverse-Distance Spatial Matrix**

| Ecological domain | Measure/variable | Counties | | | CZs | | |
| --- | --- | --- | --- | --- | --- | --- | --- |
|  |  | Moran’s I | std. err. | z-score | Moran’s I | std. err. | z-score |
| Entrepreneurial | % workforce self-employed | 0.549*** | .006 | 95.331 | 0.059*** | .015 | 3.957 |
|  | Businesses with 1-4 employees | 0.241*** | .006 | 41.803 | 0.054*** | .015 | 3.612 |
|  | % creative class | 0.353*** | .006 | 61.353 | -0.005 | .015 | -0.218 |
| Economic | Total bank deposits | 0.160*** | .006 | 28.708 | -0.017 | .015 | -1.022 |
|  | % population below poverty | 0.505*** | .006 | 87.749 | -0.021 | .015 | -1.259 |
|  | Unemployment rate | 0.367*** | .006 | 63.819 | -0.009 | .015 | -0.469 |
|  | Per capita income | 0.458*** | .006 | 79.626 | 0.016 | .015 | 1.139 |
| Social | Associations per 10,000 | 0.427*** | .006 | 74.187 | 0.008 | .015 | 0.598 |
|  | Third places per 10,000 | 0.345*** | .006 | 60.183 | 0.037 | .015 | 2.525 |
|  | Voters in 2000 | 0.399*** | .006 | 70.233 | 0.012 | .015 | 0.854 |
|  | Adherents to civic denominations | 0.701*** | .006 | 121.663 | 0.002 | .015 | 0.244 |
| Demographic | % population identify as Black | 0.724*** | .006 | 125.660 | 0.006 | .015 | 0.469 |
|  | % population identify as Hispanic | 0.784*** | .006 | 136.419 | -0.007 | .015 | -0.390 |
|  | % adult population with ≥ bachelor’s | 0.318*** | .006 | 55.328 | -0.012 | .015 | -0.712 |
|  | % population age 25 and younger | 0.267*** | .006 | 46.395 | -0.006 | .015 | -0.313 |
|  | % population age 65 and older | 0.382*** | .006 | 66.399 | 0.011 | .015 | 0.791 |
| Observations |  | 3,109 |  |  | 691 |  |  |
| Significance levels: ***<1%, **<5%, *<10% | |  |  |  |  |  |  |
| *Notes:* Table summarizes the Global Moran’s I Test for Spatial Dependence. This table uses an inverse-distance based spatial weighting matrix to produce the Moran’s I Index (displayed), Expected Index, Variance (displayed), z-score (displayed), and p-value (displayed). The weight was constructed in GeoDa 1.16, and test performed with the *SPATGSA* [62] command in Stata 17/SE. | | | | | | | |
| *Abbreviations:* Voters in 2000, percent of eligible population that vote in presidential election (2000) per 1000 population; Total bank deposits, commercial banks and savings institutions – total deposits; std. err., standard error. | | | | | | | |
