# Supplementary material for: When to use commuting zones? An empirical description of spatial autocorrelation in U.S. counties versus commuting zones
Source: PLoS One. 2022 Jul 13;17(7):e0270303. doi: 10.1371/journal.pone.0270303 (PMC9278745; doi:10.1371/journal.pone.0270303)
Supplement: S3 Table — Significance levels: ***<1%, **<5%, *<10% Notes: Table summarizes the Global Moran’s I Test for Spatial Dependence. This table uses an inverse-distance based spatial weighting matrix to produce the Moran’s I Index (displayed), Expected Index, Variance (displayed), z-score (displayed), and p-value (displayed). The weight was constructed in GeoDa 1.16, and test performed with the SPATGSA [62] command in Stata 17/SE. Abbreviations: LQs, Location Quotients; NAICS, North American Industrial Classification System; std. err., standard error. (DOCX) [file pone.0270303.s003.docx]

**S2.2 Table. Global Moran’s I for Industrial LQs (Counties versus CZs), Inverse-Distance Spatial Matrix**

| NAICS | Counties | | | CZs | | |
| --- | --- | --- | --- | --- | --- | --- |
|  | Moran’s I | std. err. | z-score | Moran’s I | std. err. | z-score |
| 11) Agriculture, Forestry, Fishing, and Hunting | 0.233*** | .006 | 40.959 | 0.049*** | .015 | 3.333 |
| 21) Mining, Quarrying, and Oil/Gas Extraction | 0.277*** | .006 | 48.632 | 0.018* | .015 | 1.280 |
| 22) Utilities | 0.045*** | .006 | 8.015 | -0.011 | .015 | -0.615 |
| 23) Construction | 0.101*** | .006 | 17.601 | -0.005 | .015 | -0.238 |
| 31-33) Manufacturing | 0.367*** | .006 | 63.650 | 0.032** | .015 | 2.136 |
| 42) Wholesale Trade | 0.202*** | .006 | 35.133 | -0.021* | .014 | -1.348 |
| 44-45) Retail Trade | 0.118*** | .006 | 20.477 | 0.025** | .015 | 1.709 |
| 48-49) Transportation and Warehousing | 0.032*** | .006 | 5.723 | 0.027** | .015 | 1.886 |
| 51) Information | 0.053*** | .006 | 9.296 | -0.007 | .015 | -0.349 |
| 52) Finance and Insurance | 0.132*** | .006 | 23.168 | 0.017 | .015 | 1.230 |
| 53) Real Estate and Rental and Leasing | 0.086*** | .005 | 15.803 | 0.008 | .015 | 0.651 |
| 54) Professional, Scientific, and Technical Services | 0.178*** | .006 | 31.252 | 0.004 | .015 | 0.371 |
| 55) Management of Companies and Enterprises | 0.072*** | .006 | 12.635 | 0.044*** | .015 | 3.008 |
| 56) Administrative, Support, Waste Management | 0.121*** | .006 | 21.225 | 0.019* | .015 | 1.361 |
| 61) Educational Services | 0.038*** | .006 | 6.789 | -0.022* | .014 | -1.393 |
| 62) Health Care and Social Assistance | 0.141*** | .006 | 24.446 | -0.009 | .015 | -0.485 |
| 71) Arts, Entertainment, and Recreation | 0.087*** | .006 | 15.689 | -0.001 | .015 | 0.008 |
| 72) Accommodation and Food Services | 0.186*** | .006 | 32.470 | 0.025 | .015 | 1.702 |
| 81) Other Services | 0.132*** | .006 | 22.952 | 0.016 | .015 | 1.135 |
| Observations | 3,109 |  |  | 691 |  |  |
| Significance levels: ***<1%, **<5%, *<10% |  |  |  |  |  |  |
| *Notes:* Table summarizes the Global Moran’s I Test for Spatial Dependence. This table uses a inverse-distance based spatial weighting matrix to produce the Moran’s I Index (displayed), Expected Index, Variance (displayed), z-score (displayed), and p-value (displayed). The weight was constructed in GeoDa 1.16, and test performed with the *SPATGSA* [62] command in Stata 17/SE. | | | | | | |
| *Abbreviations:* LQs, Location Quotients; NAICS, North American Industrial Classification System; std. err., standard error. | | | | | | |
