# Supplementary material for: When to use commuting zones? An empirical description of spatial autocorrelation in U.S. counties versus commuting zones
Source: PLoS One. 2022 Jul 13;17(7):e0270303. doi: 10.1371/journal.pone.0270303 (PMC9278745; doi:10.1371/journal.pone.0270303)
Supplement: S4 Table — Significance levels: ***<1%, **<5%, *<10% Notes: Table summarizes the Global Geary’s c Test for Spatial Autocorrelation. This table uses an inverse-distance based spatial weighting matrix to produce Geary’s contiguity ratio [0,2] (displayed), Variance (displayed), z-score (displayed), and p-value (displayed). The weight was constructed in GeoDa 1.16, and test performed with the SPATGSA [62] command in Stata 17/SE. Abbreviations: Voters in 2000, percent of eligible population that vote in presidential election (2000) per 1000 population; Total bank deposits, commercial banks and savings institutions—total deposits; std. err., standard error. (DOCX) [file pone.0270303.s004.docx]

**S3.1 Table. Global Geary’s c for Entrepreneurial, Economic, Social, and Demographic Domains (Counties versus CZs), Inverse-Distance Spatial Matrix**

| Ecological domain | Measure/variable | Counties | | | CZs | | |
| --- | --- | --- | --- | --- | --- | --- | --- |
|  |  | Geary’s c | std. err. | z-score | Geary’s c | std. err. | z-score |
| Entrepreneurial | % workforce self-employed | 0.447*** | .007 | -83.989 | 0.939*** | .017 | -3.529 |
|  | Businesses with 1-4 employees | 0.762*** | .006 | -37.191 | 0.940*** | .016 | -3.682 |
|  | % creative class | 0.666*** | .006 | -52.621 | 1.010 | .016 | 0.603 |
| Economic | Total bank deposits | 0.859*** | .017 | -8.307 | 1.044* | .025 | 1.779 |
|  | % population below poverty | 0.492*** | .006 | -80.505 | 1.021 | .017 | 1.208 |
|  | Unemployment rate | 0.631*** | .007 | -54.684 | 1.011 | .018 | 0.615 |
|  | Per capita income | 0.552*** | .007 | -67.878 | 0.985 | .017 | -0.912 |
| Social | Associations per 10,000 | 0.577*** | .006 | -66.031 | 0.998 | .017 | -0.133 |
|  | Third places per 10,000 | 0.658*** | .009 | -38.879 | 0.976 | .020 | -1.223 |
|  | Voters in 2000 | 0.607*** | .013 | -31.291 | 0.985 | .016 | -0.951 |
|  | Adherents to civic denominations | 0.302*** | .006 | -115.559 | 1.000 | .016 | -0.019 |
| Demographic | % population identify as Black | 0.284*** | .007 | -110.128 | 0.991 | .017 | -0.525 |
|  | % population identify as Hispanic | 0.206*** | .008 | -103.079 | 0.989 | .019 | -0.558 |
|  | % adult population with ≥ bachelor’s | 0.701*** | .006 | -46.430 | 1.016 | .016 | 0.966 |
|  | % population age 25 and younger | 0.734*** | .006 | -41.482 | 1.007 | .017 | 0.388 |
|  | % population age 65 and older | 0.618*** | .006 | -62.667 | 0.990 | .016 | -0.644 |
| Observations |  | 3,109 |  |  | 691 |  |  |
| Significance levels: ***<1%, **<5%, *<10% | |  |  |  |  |  |  |
| *Notes:* Table summarizes the Global Geary’s c Test for Spatial Autocorrelation. This table uses an inverse-distance based spatial weighting matrix to produce Geary’s contiguity ratio [0,2] (displayed), Variance (displayed), z-score (displayed), and p-value (displayed). The weight was constructed in GeoDa 1.16, and test performed with the *SPATGSA* [62] command in Stata 17/SE. | | | | | | | |
| *Abbreviations:* Voters in 2000, percent of eligible population that vote in presidential election (2000) per 1000 population; Total bank deposits, commercial banks and savings institutions – total deposits; std. err., standard error. | | | | | | | |
