# Supplementary material for: When to use commuting zones? An empirical description of spatial autocorrelation in U.S. counties versus commuting zones
Source: PLoS One. 2022 Jul 13;17(7):e0270303. doi: 10.1371/journal.pone.0270303 (PMC9278745; doi:10.1371/journal.pone.0270303)
Supplement: S5 Table — Significance levels: ***<1%, **<5%, *<10% Notes: Table summarizes the Global Geary’s c Test for Spatial Autocorrelation. This table uses an inverse-distance based spatial weighting matrix to produce Geary’s contiguity ratio [0,2] (displayed), Variance (displayed), z-score (displayed), and p-value (displayed). The weight was constructed in GeoDa 1.16, and test performed with the SPATGSA [62] command in Stata 17/SE. Abbreviations: LQs, Location Quotients; NAICS, North American Industrial Classification System; std. err., standard error. (DOCX) [file pone.0270303.s005.docx]

**S3.2 Table Global Geary’s c for Industrial LQs (Counties versus CZs), Inverse-Distance Spatial Matrix**

| NAICS | Counties | | | CZs | | |
| --- | --- | --- | --- | --- | --- | --- |
|  | Geary’s c | std. err. | z-score | Geary’s c | std. err. | z-score |
| 11) Agriculture, Forestry, Fishing, and Hunting | 0.772*** | .011 | -20.131 | 0.941*** | .024 | -2.450 |
| 21) Mining, Quarrying, and Oil/Gas Extraction | 0.786*** | .011 | -18.854 | 0.975 | .023 | -1.129 |
| 22) Utilities | 0.966* | .015 | -2.293 | 1.006 | .024 | 0.235 |
| 23) Construction | 0.913*** | .008 | -10.460 | 0.995 | .020 | -0.266 |
| 31-33) Manufacturing | 0.635*** | .006 | -60.818 | 0.967** | .016 | -2.050 |
| 42) Wholesale Trade | 0.804*** | .008 | -25.432 | 1.031 | .031 | 1.001 |
| 44-45) Retail Trade | 0.890*** | .006 | -17.133 | 0.991 | .018 | -0.497 |
| 48-49) Transportation and Warehousing | 0.975* | .016 | -1.548 | 0.977 | .022 | -1.047 |
| 51) Information | 0.939*** | .011 | -5.640 | 1.005 | .020 | 0.232 |
| 52) Finance and Insurance | 0.886 | .011 | -10.040 | 0.998 | .021 | -0.084 |
| 53) Real Estate and Rental and Leasing | 0.911*** | .022 | -3.989 | 0.993 | .021 | -0.322 |
| 54) Professional, Scientific, and Technical Services | 0.854*** | .012 | -12.712 | 1.001 | .018 | 0.075 |
| 55) Management of Companies and Enterprises | 0.943*** | .011 | -5.291 | 0.902*** | .020 | -4.834 |
| 56) Administrative, Support, Waste Management | 0.895*** | .009 | -11.810 | 0.980 | .021 | -0.944 |
| 61) Educational Services | 0.970*** | .011 | -2.720 | 1.031 | .032 | 0.974 |
| 62) Health Care and Social Assistance | 0.866*** | .007 | -20.294 | 1.017 | .017 | 0.990 |
| 71) Arts, Entertainment, and Recreation | 0.954*** | .018 | -2.524 | 0.995 | .028 | -0.171 |
| 72) Accommodation and Food Services | 0.805*** | .008 | -23.304 | 0.978 | .019 | -1.178 |
| 81) Other Services | 0.873*** | .008 | -16.869 | 0.985 | .021 | -0.697 |
| Observations | 3,109 |  |  | 691 |  |  |
| Significance levels: ***<1%, **<5%, *<10% |  |  |  |  |  |  |
| *Notes:* Table summarizes the Global Geary’s c Test for Spatial Autocorrelation. This table uses an inverse-distance based spatial weighting matrix to produce Geary’s contiguity ratio [0,2] (displayed), Variance (displayed), z-score (displayed), and p-value (displayed). The weight was constructed in GeoDa 1.16, and test performed with the *SPATGSA* [62] command in Stata 17/SE. | | | | | | |
| *Abbreviations:* LQs, Location Quotients; NAICS, North American Industrial Classification System; std. err., standard error. | | | | | | |
