# Supplementary material for: When to use commuting zones? An empirical description of spatial autocorrelation in U.S. counties versus commuting zones
Source: PLoS One. 2022 Jul 13;17(7):e0270303. doi: 10.1371/journal.pone.0270303 (PMC9278745; doi:10.1371/journal.pone.0270303)
Supplement: S1 Fig — (PDF) [file pone.0270303.s006.pdf]

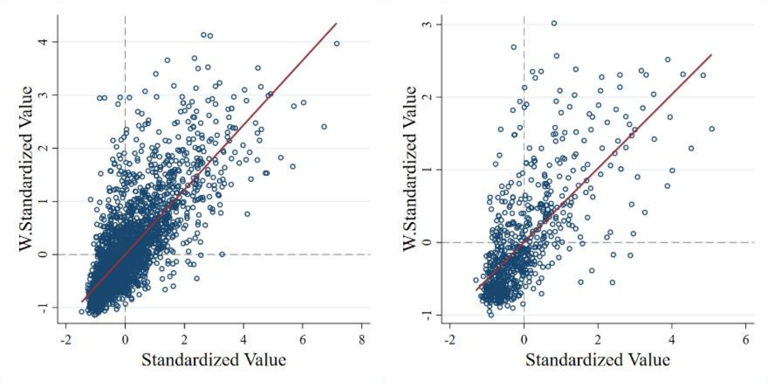

Percent workforce self-employed

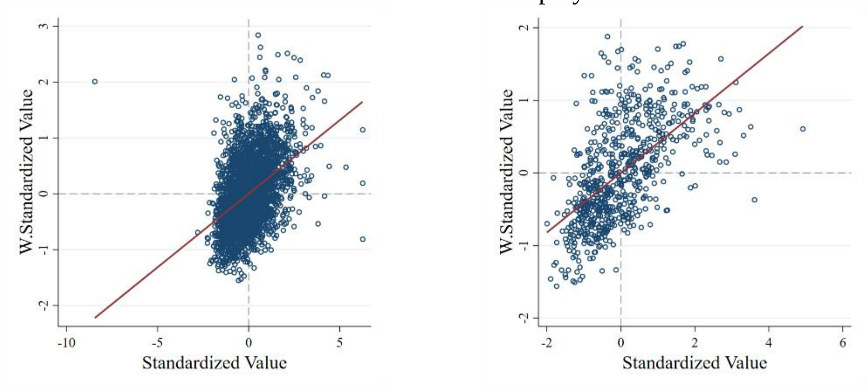

Percent businesses with 1-4 employees

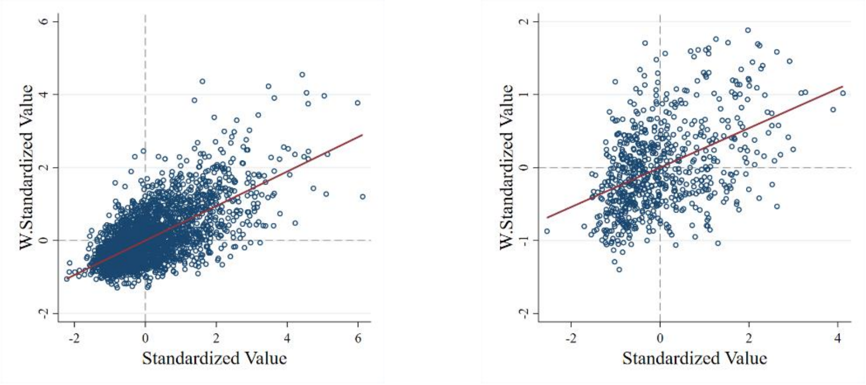

Percent population creative class

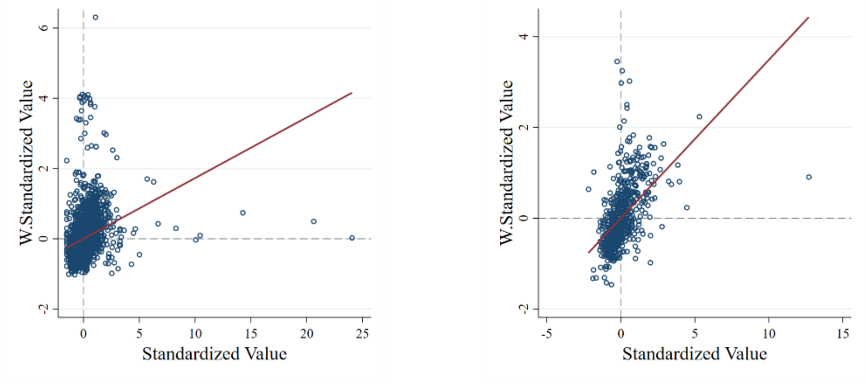

Total bank deposits

S1 Figure. Moran Scatter Plot for Entrepreneurship Variables (counties left, CZs right)
