# Supplementary figures and images for: When to use commuting zones? An empirical description of spatial autocorrelation in U.S. counties versus commuting zones
Source: PLoS One. 2022 Jul 13;17(7):e0270303. doi: 10.1371/journal.pone.0270303 (PMC9278745; doi:10.1371/journal.pone.0270303)

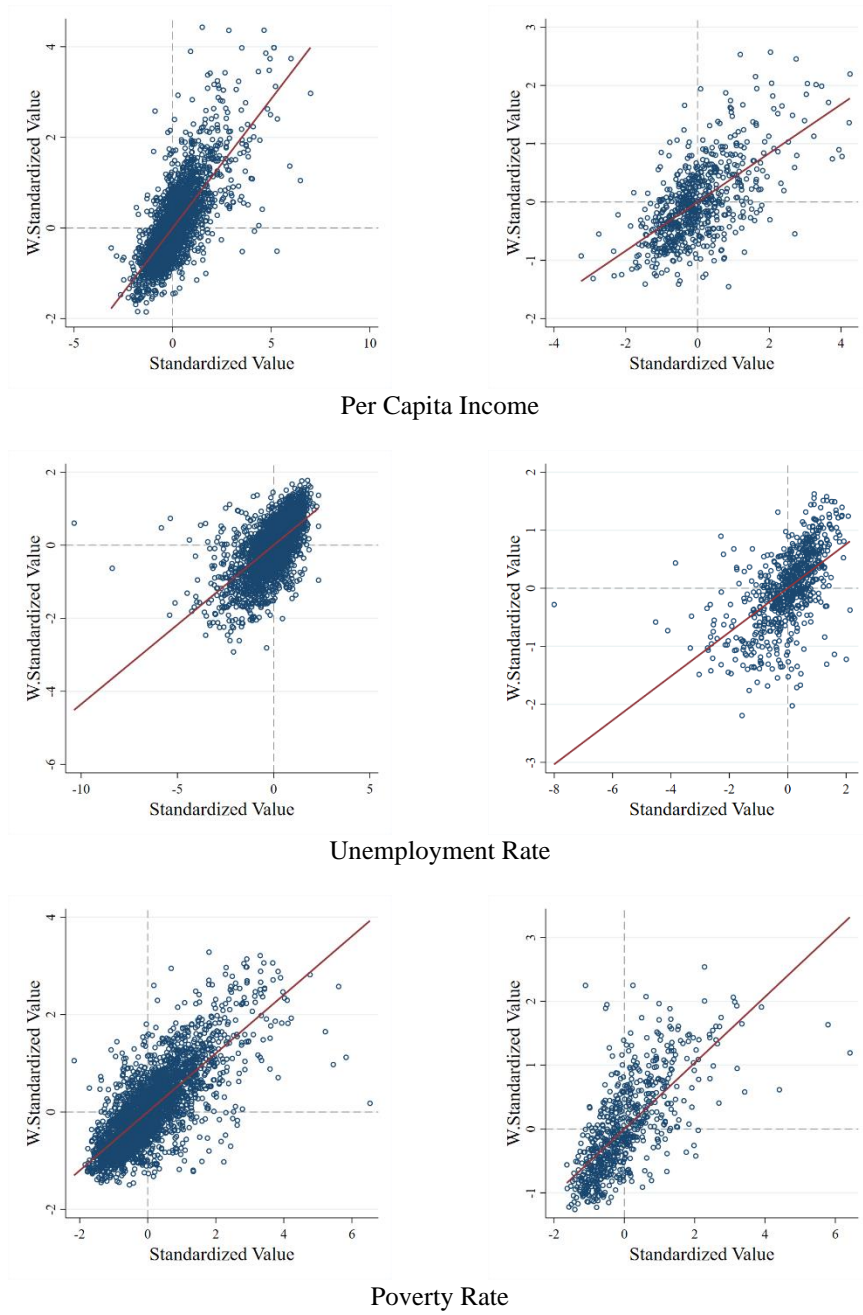

**S2 Figure. Moran Scatter Plot for Economic and Social Variables (counties left, CZs right)**

Supplement: S2 Fig — (PDF) [file pone.0270303.s007.pdf]

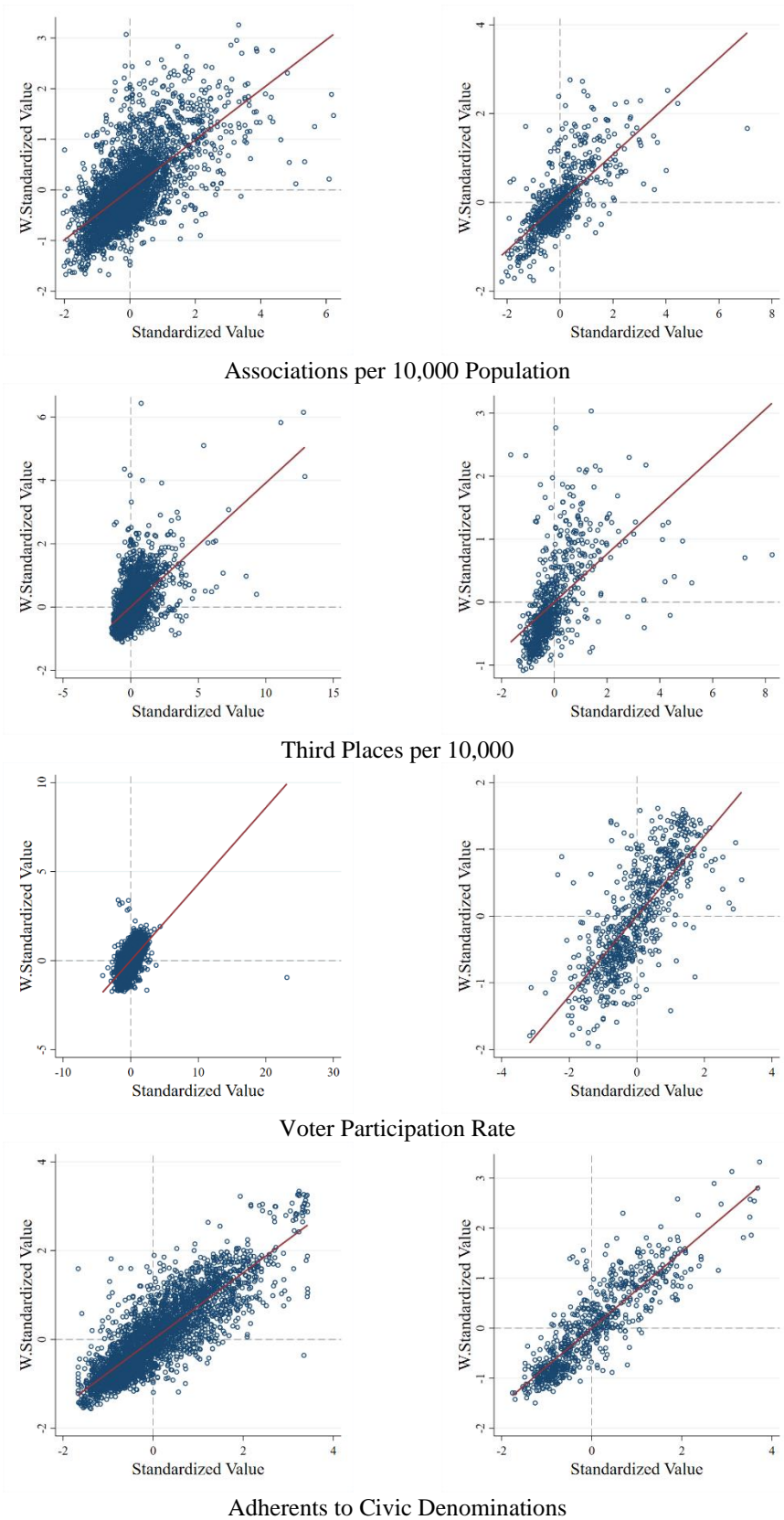

**S3 Figure. Moran Scatter Plot for Social Variables (counties left, CZs right)**

Supplement: S3 Fig — (PDF) [file pone.0270303.s008.pdf]
