# Supplementary material for: When to use commuting zones? An empirical description of spatial autocorrelation in U.S. counties versus commuting zones
Source: PLoS One. 2022 Jul 13;17(7):e0270303. doi: 10.1371/journal.pone.0270303 (PMC9278745; doi:10.1371/journal.pone.0270303)
Supplement: S4 Fig — (PDF) [file pone.0270303.s009.pdf]

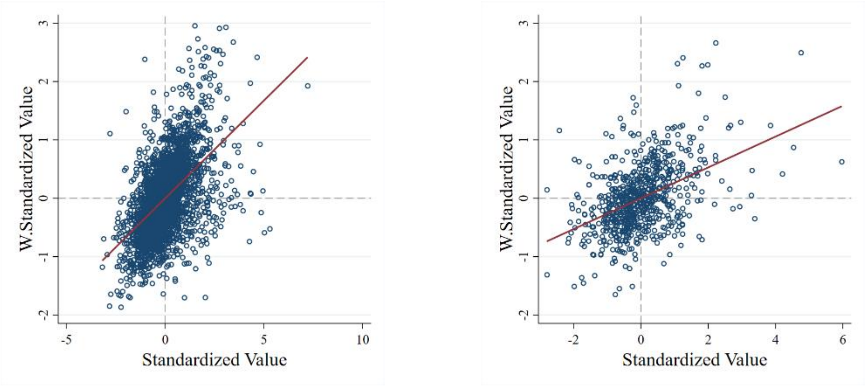

Percent of the Population Age 25 or Younger

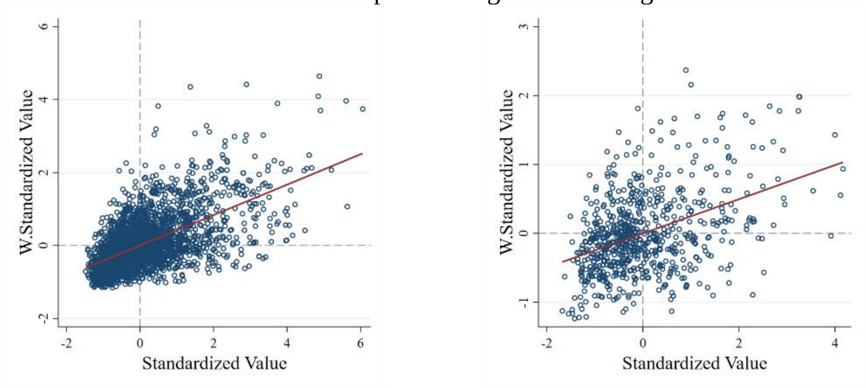

Percent of the Population with a Bachelor's Degree

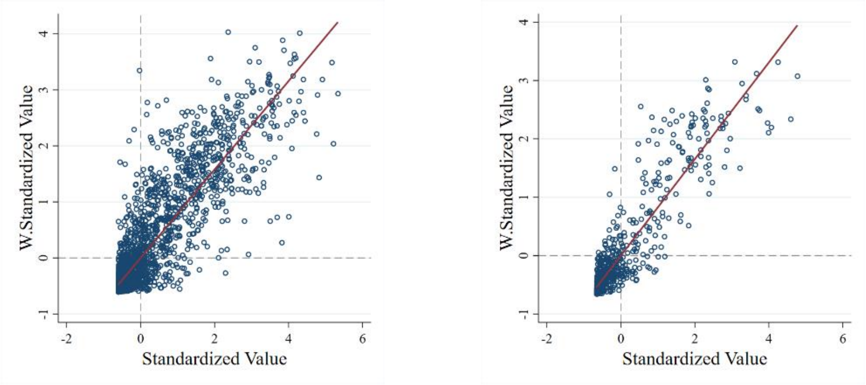

Percent of the Population Identifying as Black

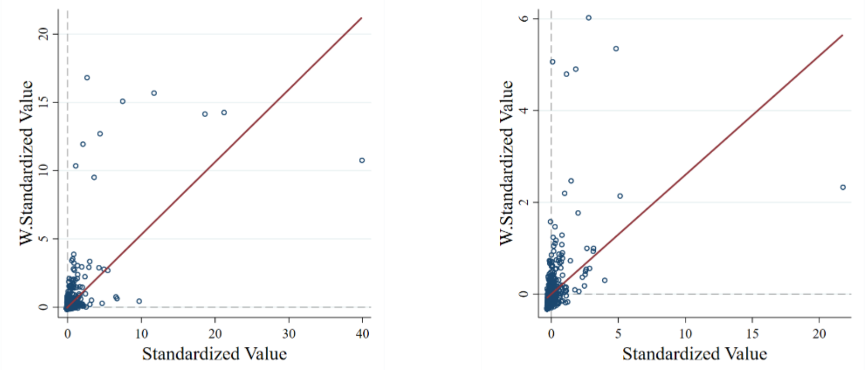

Population Density

S4 Figure. Moran Scatter Plot for Demographic Variables (counties left, CZs right)
