# Supplementary material for: When to use commuting zones? An empirical description of spatial autocorrelation in U.S. counties versus commuting zones
Source: PLoS One. 2022 Jul 13;17(7):e0270303. doi: 10.1371/journal.pone.0270303 (PMC9278745; doi:10.1371/journal.pone.0270303)
Supplement: S5 Fig — (PDF) [file pone.0270303.s010.pdf]

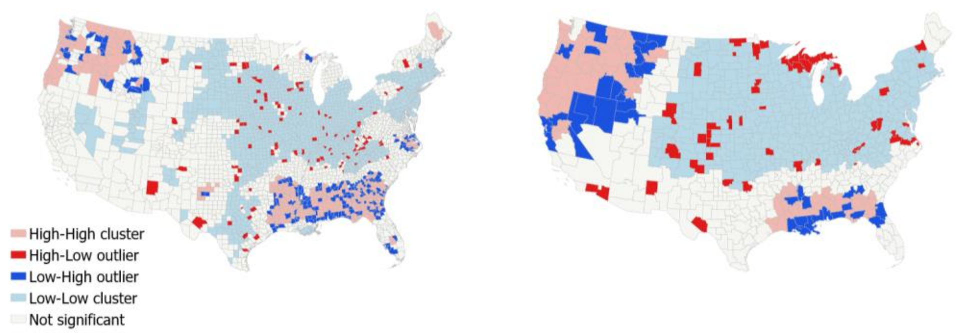

(NAICS 11) Ag, Forestry, etc.

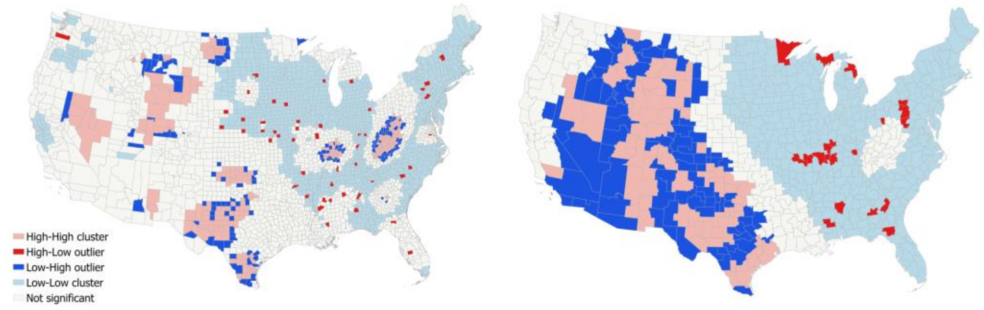

(NAICS 21) Mining, Quarrying, Oil, etc.

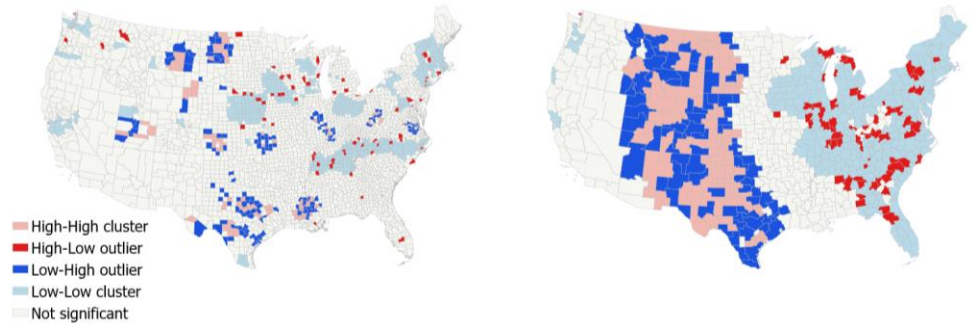

(NAICS 22) Utilities

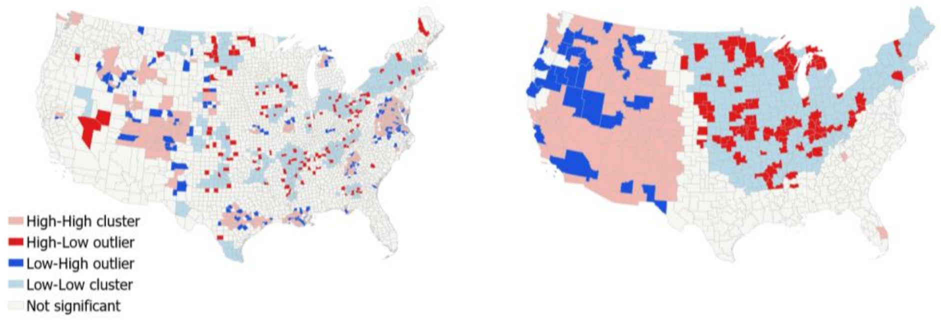

(NAICS 23) Construction

S5 Figure. LISA Cluster Map for Industrial LQs (counties left, CZs right)

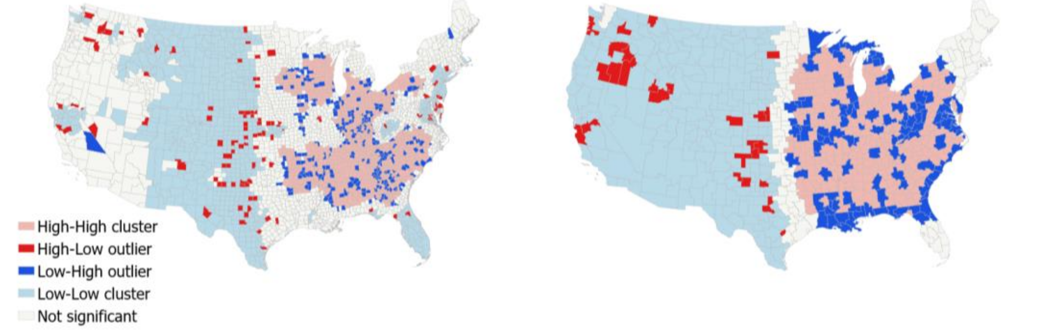

(NAICS 31-33) Manufacturing

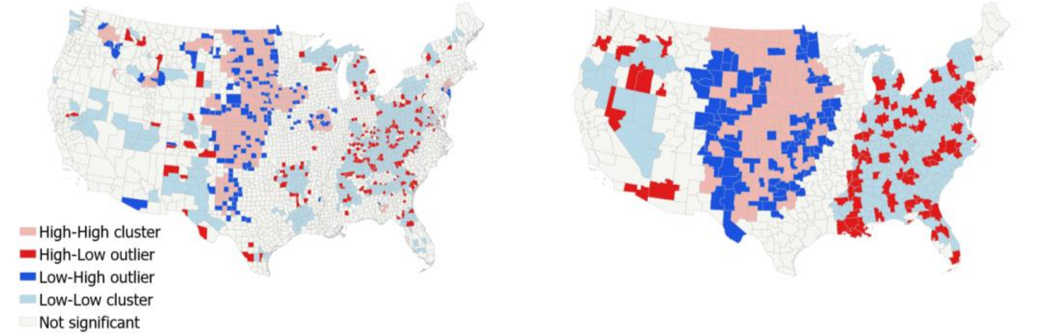

(NAICS 42) Wholesale Trade

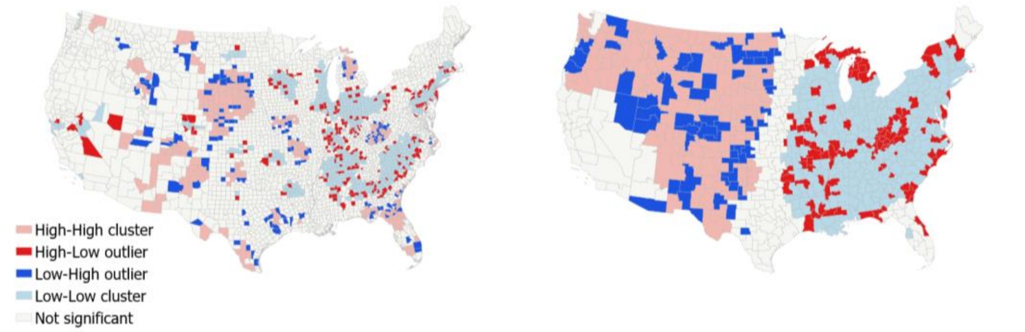

(NAICS 44-45) Retail Trade

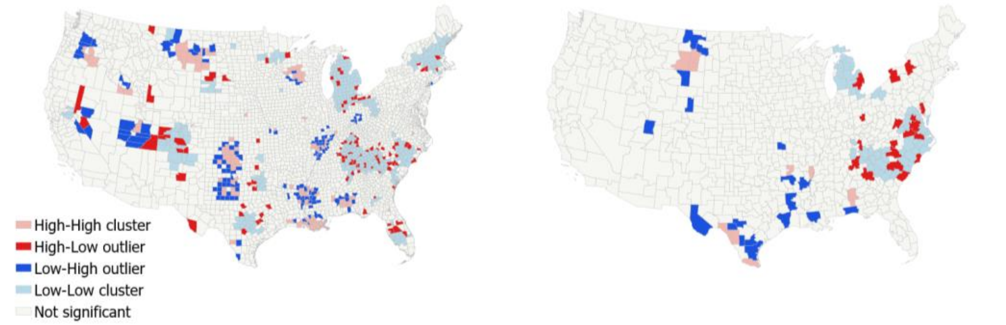

(NAICS 48-49) Transportation and Warehousing

S5 Figure (cont). LISA Cluster Map for Industrial LQs (counties left, CZs right)

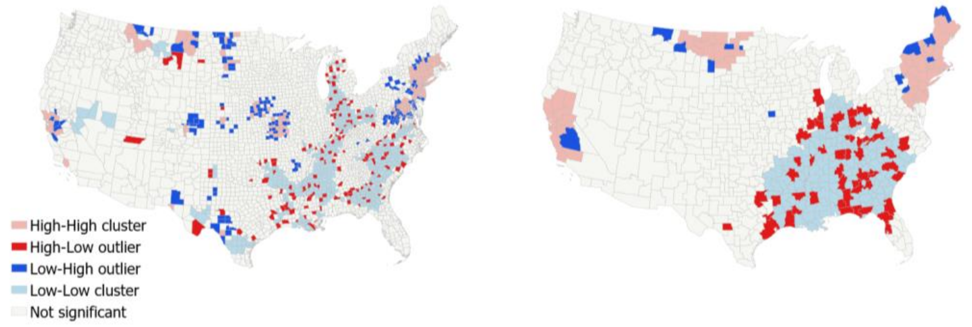

(NAICS 51) Information

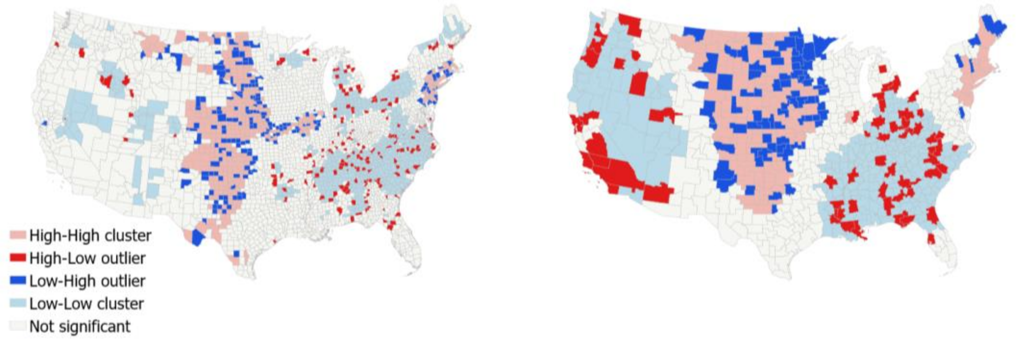

(NAICS 52) Finance and Insurance

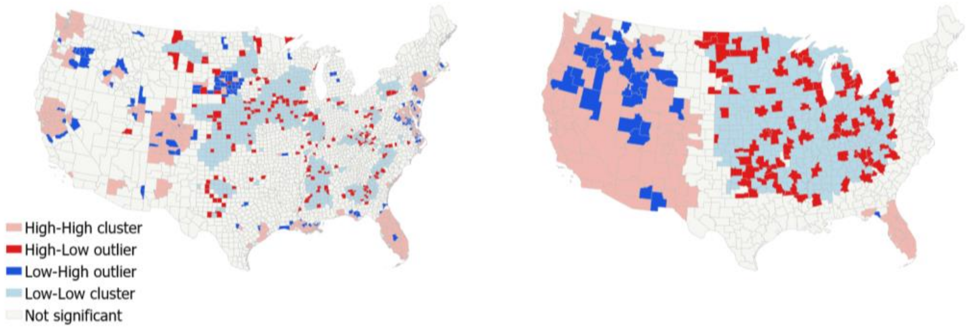

(NAICS 53) Real Estate and Rental and Leasing

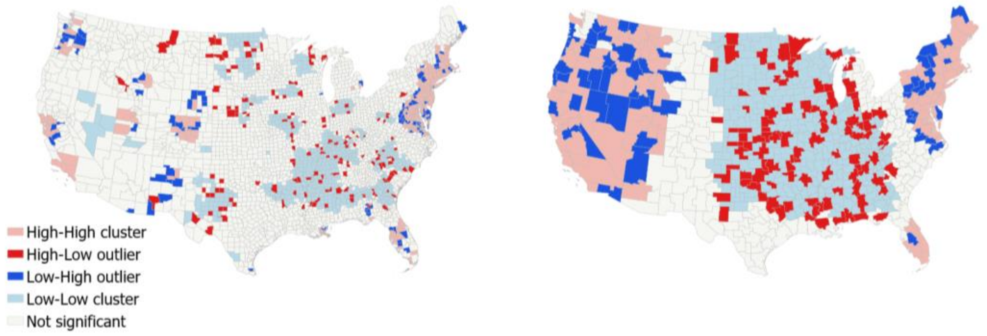

(NAICS 54) Prof., Scientific, and Tech. Services

S5 Figure (cont). LISA Cluster Map for Industrial LQs (counties left, CZs right)

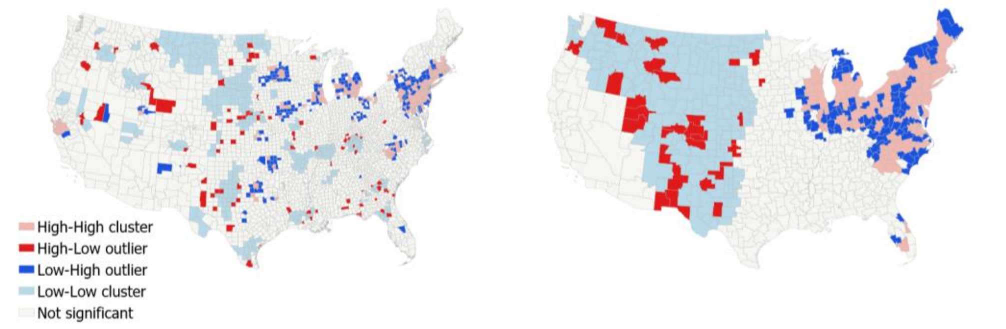

(NAICS 55) Management of Employees

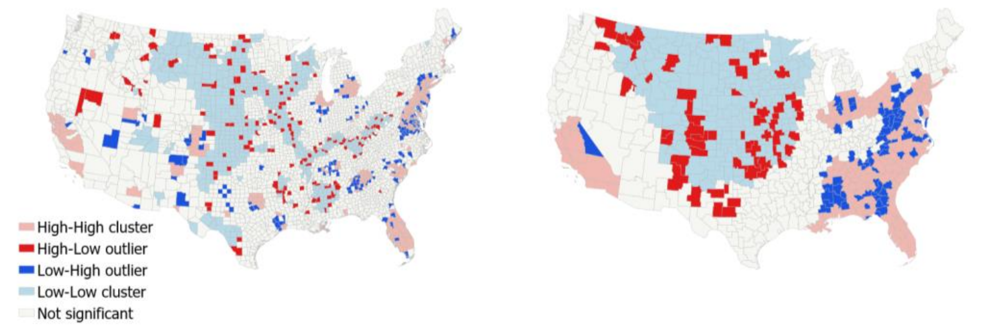

(NAICS 56) Admin., Support, Waste Management, etc.

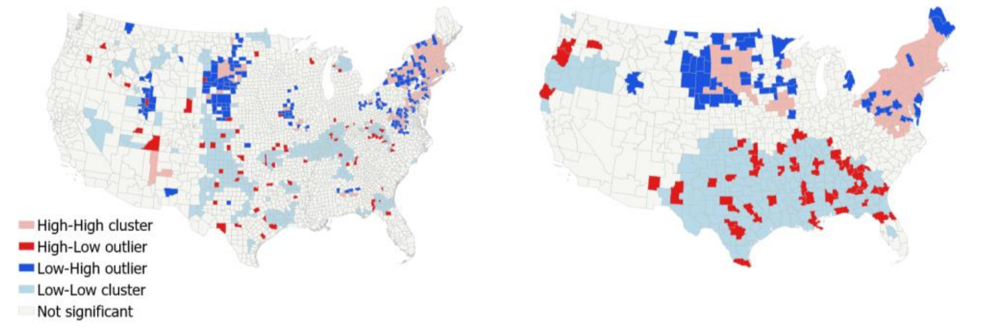

(NAICS 61) Educational Services

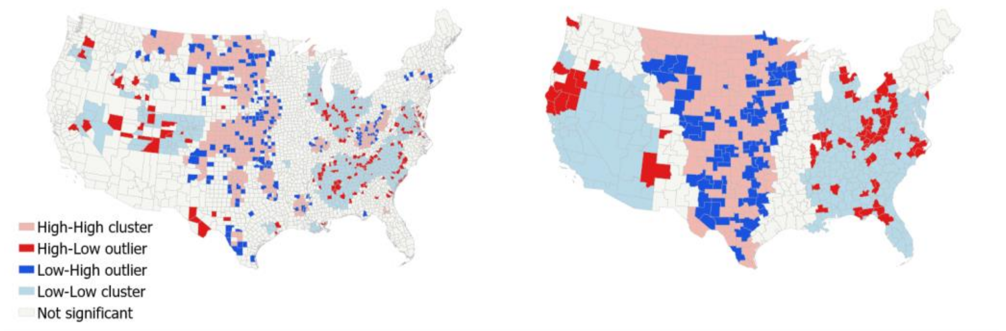

(NAICS 62) Health Care and Social Assistance

S5 Figure (cont). LISA Cluster Map for Industrial LQs (counties left, CZs right)

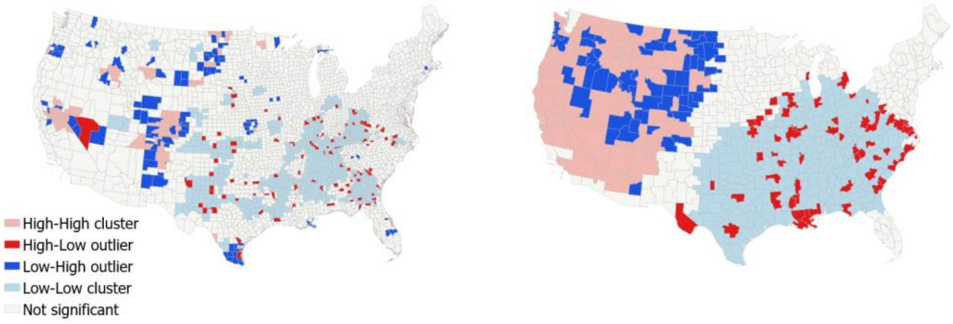

(NAICS 71) Arts, Entertainment, and Recreation

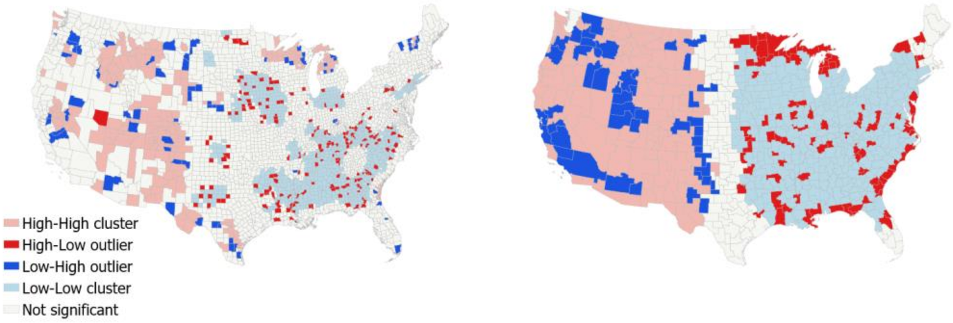

(NAICS 72) Accommodation and Food Services

S5 Figure (cont). LISA Cluster Map for Industrial LQs (counties left, CZs right)
