# Supplementary material for: When to use commuting zones? An empirical description of spatial autocorrelation in U.S. counties versus commuting zones
Source: PLoS One. 2022 Jul 13;17(7):e0270303. doi: 10.1371/journal.pone.0270303 (PMC9278745; doi:10.1371/journal.pone.0270303)
Supplement: S7 Fig — (PDF) [file pone.0270303.s012.pdf]

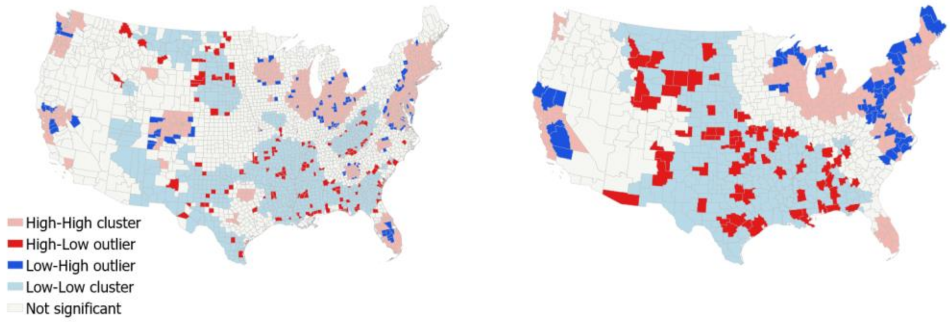

Per Capita Income

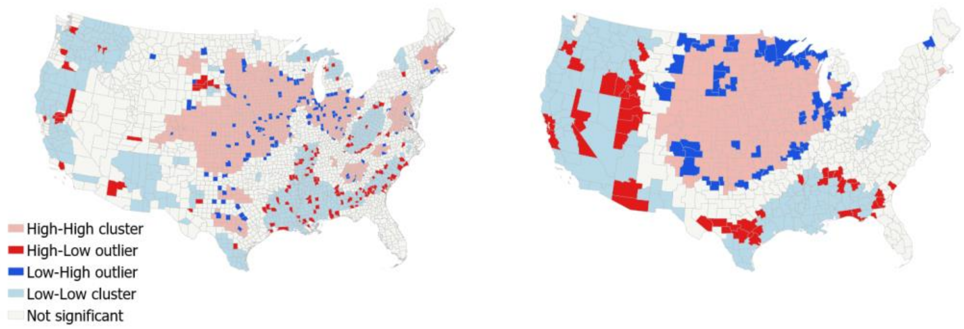

Unemployment Rate

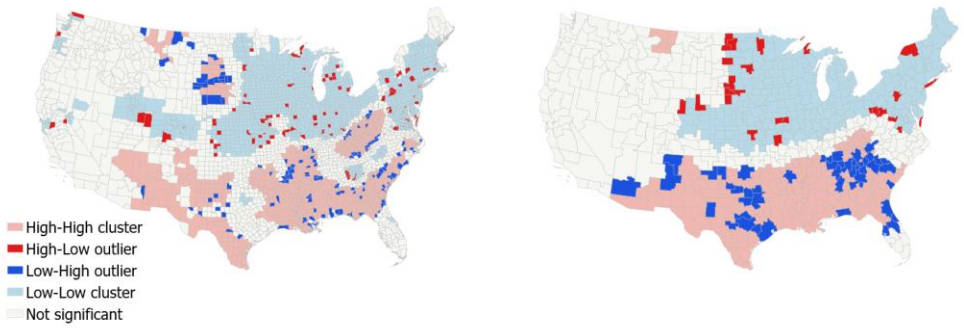

Poverty Rate

S7 Figure. LISA Cluster Map for Economic Variables (counties left, CZs right)
