# Supplementary material for: When to use commuting zones? An empirical description of spatial autocorrelation in U.S. counties versus commuting zones
Source: PLoS One. 2022 Jul 13;17(7):e0270303. doi: 10.1371/journal.pone.0270303 (PMC9278745; doi:10.1371/journal.pone.0270303)
Supplement: S9 Fig — (PDF) [file pone.0270303.s014.pdf]

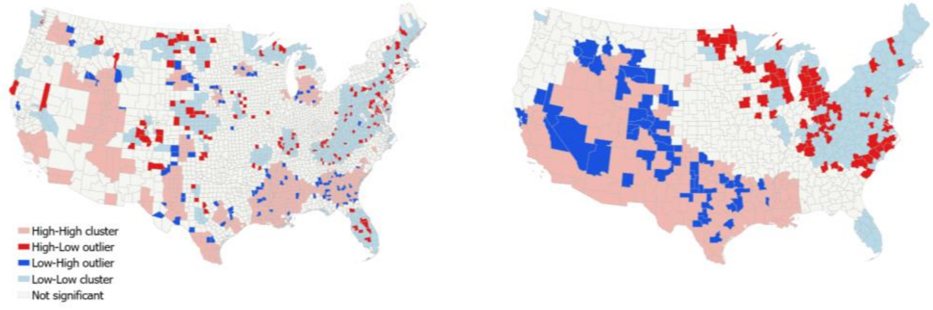

Percent of the Population Age 25 or Younger

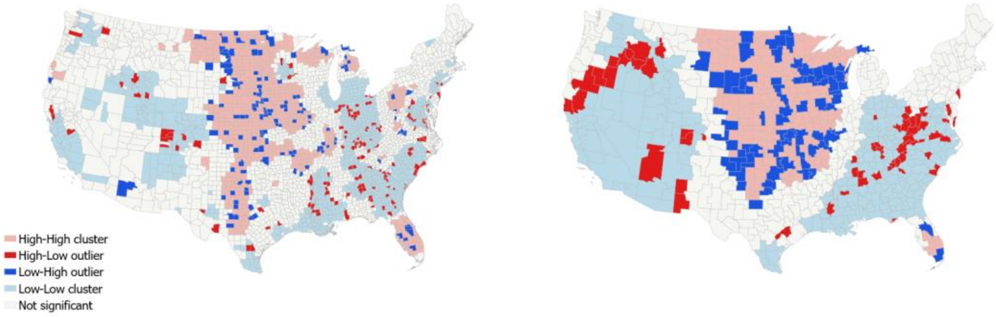

Percent of the Population Age 65 or Older

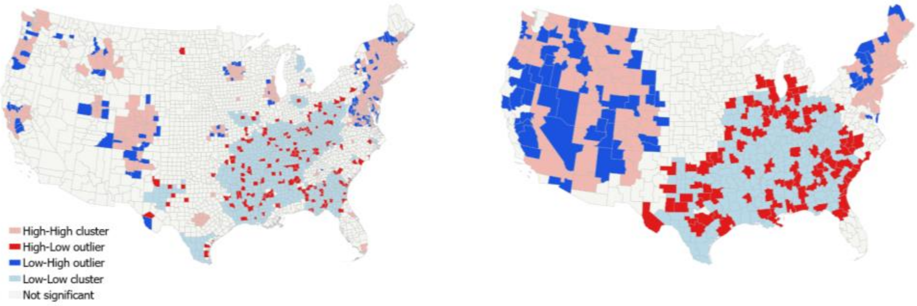

Percent of the Population with a Bachelor's Degree

S9 Figure. LISA Cluster Map for Demographic Variables (counties left, CZs right)

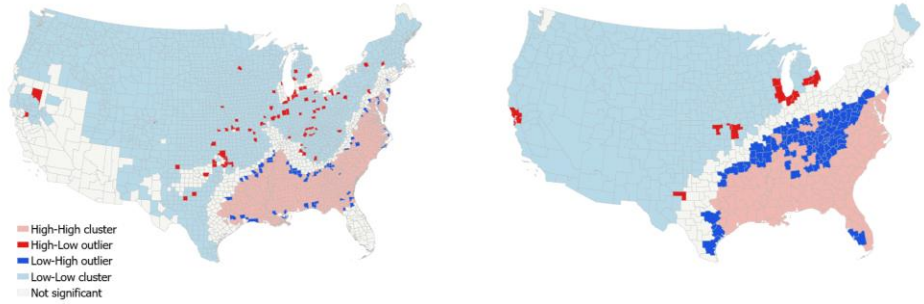

Percent of the Population Identifying as Black

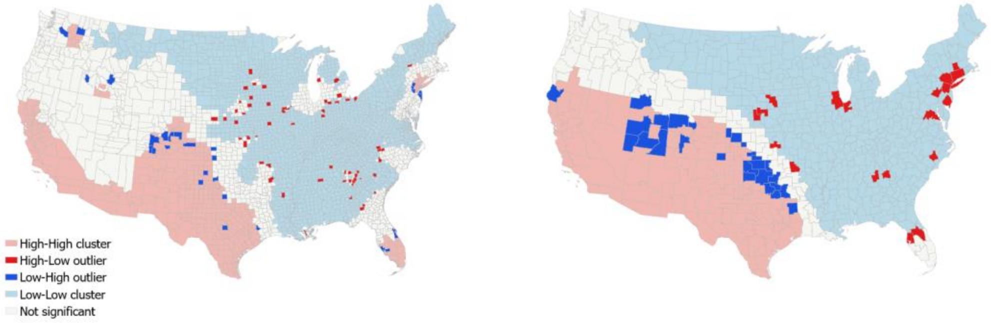

Percent of the Population Identifying as Hispanic

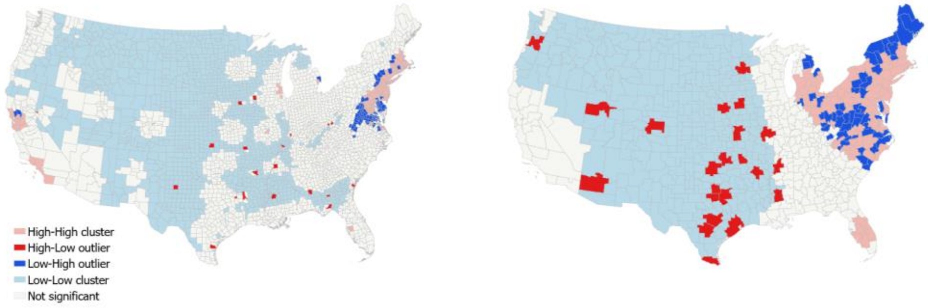

Population Density

S9 Figure (cont). LISA Cluster Map for Demographic Variables (counties left, CZs right)
