# Supplementary material for: When to use commuting zones? An empirical description of spatial autocorrelation in U.S. counties versus commuting zones
Source: PLoS One. 2022 Jul 13;17(7):e0270303. doi: 10.1371/journal.pone.0270303 (PMC9278745; doi:10.1371/journal.pone.0270303)
Supplement: Attachment — Submitted filename: Review report on PONE-D-21-36293.pdf [file pone.0270303.s015.pdf]

Review report on PONE-D-21-36293

Overview:

This paper calculated Local Moran's I index for various regional variables measured at the community or commuting zone (CZ) level. They found that CZs reduce – but do not eliminate – spatial dependence for most variables across categories. I have some major concerns about this paper, those listed as follows.

Major concerns:

1) I think this paper is not up to the standard of international journals. The results are presented in just 16 lines of text and there is no discussion of the results. The explanation is poor and for instance, the definition of "High-High cluster" or "High-Low outlier" are not explained although they are main results.
2) Table 2.1 shows the result of calculating Moran's I-index. The value is too high. The original Moran's I-index takes values from [-1 to 1] when W is row-standardized. Therefore, I believe that the values shown in this table are the standardized Moran's I-index (z-values). However, this value is too high compared to the sample size (in fact, the sample size itself is not clear). Since there is no clear explanation, I could not understand what this value represents. In the explanation under Table 2.1, you explained that it was a chi-square value, but usually, normal distribution permutation test is used for the inference of Moran's I. See Anselin (1995).
3) It is well known in the MAUP literature that the results of Moran's I depend on spatial scale. Do the results in your paper add new evidence to the existing knowledge base? I think literature review may be improved by reflecting such perspective.
4) This paper calculated Moran's I index at different spatial scales. However, I could not understand what you wanted to discuss in this comparison. For example, suppose we have a situation where a CZ is classified as High-Low, but a community belonging to the CZ is classified as High-High. How should the readers of your paper interpret such a result?

Specific comments

1. P.5 L.91: x_i may be $(x_i - \bar{x})$. Please confirm the definition of Moran's *I*.
2. P.5 L.96: Moran's I does not presuppose homogeneity. The terms homogeneity, heterogeneity and spillover in this paper are vague and difficult to understand.
3. P.5 L.105 Anselin (1993) may be Anselin (1996).
4. Please add descriptive statistics.
5. Please add the definition of significance code (for instance, *** in table 2.1).
6. Please be considerate of non-US readers.
