# Supplementary material for: When to use commuting zones? An empirical description of spatial autocorrelation in U.S. counties versus commuting zones
Source: PLoS One. 2022 Jul 13;17(7):e0270303. doi: 10.1371/journal.pone.0270303 (PMC9278745; doi:10.1371/journal.pone.0270303)
Supplement: Attachment — Submitted filename: Response to Reviewers.docx [file pone.0270303.s017.docx]

When to Use U.S. Commuting Zones? An Empirical Description of Spatial Autocorrelation in U.S. Counties versus Commuting Zones

Response to Reviewers

# Editor Comments

Thank you for submitting your revised manuscript to PLOS ONE. After careful consideration, we feel that it has merit but does not fully meet PLOS ONE’s publication criteria as it currently stands. Therefore, we invite you to submit a revised version of the manuscript that addresses the points raised during the review process.

Reviewer 2 raised questions on a methodological issue while the reviewer also pointed out that the results do not well support the conclusion. I expect the authors to improve the manuscript by addressing the comments.

*Thank you for the opportunity to revise and resubmit our article. We are grateful for your guidance and the reviewers’ the detailed and specific comments. We paste the reviewer and technical comments below, bulleting our respective responses and edits below each comment. We find the article improved as a result of these changes.*

# Reviewer Comments

## Reviewer #1

1. All comments have been addressed.
2. Although I suggested the rejection at the first stage, you responded well to my concerns. Therefore, I have no further comments.
   - *We reiterate our thanks to this reviewer for reviewing our article again, and for their specific and constructive comments in the previous round of reviews. We find the article much improved as a result.*

## Reviewer #2

1. I confirmed the revised version of the manuscript and could find that my suggestions were largely reflected. However, I have the following concerns or questions after reading the revised manuscript. The authors wrote that they used the Moran test for spatial dependence (chi-squared) when examining the statistical significance of the calculated values of local Moran's I and Geary's C in the footnotes of the tables (e.g., Table 2.1). However, as Reviewer #1 also pointed out, the statistical test for these local indices generally relies on Z-test rather than the chi-squared test (at least, I have never seen the use of the chi-squared test in any article).
   - *We thank the reviewer for their previous comments on this article.*
   - *‘Chi-squared’, as indicated in the footnote of the tables, was a typographic error. We updated all table footnotes to indicate that the table summarizes a test for spatial dependence or spatial autocorrelation, with significance based on the z-score.*
2. Since there is no information on, for example, the formulation of test statistics, I could not judge if the obtained results were certain. Although the authors replied that they have replaced the values with [-1, 1] values and used the suggested usual method for inference (significance), I am not sure if the correction was conducted properly as far as I checked the footnotes for the tables.
   - *We added a sentence in the table footnotes with more detail on the statistical program used to make these calculations. We indicate that “the weight was constructed in GeoDa 1.16, and test performed with the SPATGSA (Pisati, 2001) command in Stata 17/SE.”*
   - *Further, we indicate every time that that Global Geary’s c test is used that the contiguity ratio ranges from 0 to 2.*
3. Also, the values of test statistics or standard error in addition to calculated local Moran's and Geary's C should be provided. Only showing significance code does not provide sufficient information, I think.
   - *Following the reviewer’s suggestion, we added standard errors and z-scores to these tables.*
     i. *The original package (SpaceStat) did not support the production of standard error or z-scores for the Global Geary’s c test. To ensure homogeneity throughout the paper and ease replicability, we retained the weights and replaced SpaceStat with an ado package (*SPATGSA) *that provided test statistics and standard error for both the Global Moran’s I and Global Geary’s c. While some variation in the output has arisen, it does not change the outcome of the paper.*
     ii. *We were also reminded through this process that contiguity-based spatial weighting matrices were standard in the literature for county-level analysis and switched these tables with the inverse-distance based tables in the Appendix.*
4. (By the way, it seems that the authors used the sentence "Table summarizes the significance of the Moran test for spatial dependence" for the results of local Geary's C by mistake in Table 3.1 and Table 3.2, for example.)
   - *We thank the reviewer for catching this typographical error. We have fixed it.*
5. The authors concluded that CZs do indeed reduce the severity of spatial autocorrelation. However, as far as I checked the tables in the main text, I am not sure if this story consistently holds. For several ecological variables and LQs, the values of local Moran's I and Geary's C for CZs are larger than those for counties. And even if the local indices for CZs are smaller than those for counties, it is difficult to judge whether the difference between the local indices for counties and that for CZs is notable. I suggest that the authors reconsider the conclusion (and thus the structure of their manuscript) if they cannot provide a clear explanation for these matters.
   - *This is a valuable point by the reviewer and we are grateful for it. We agree that our previous conclusions were too broad and have changed the language (and added some specificity around variables) to tone down the generalizability of the conclusions.*
   - *While the results do indicate that it is more likely to see reduced spatial autocorrelation when moving to CZs, there are a number of important exceptions that the reviewer correctly points out, and the reviewer is correct that in some cases, it is difficult to judge whether the difference between the indices is notable. We added these important caveats to our conclusions and abstract.*
   - *The abstract now reads: “U.S. Commuting Zones (CZs) are an aggregation of county-level data that researchers commonly use to create less arbitrary spatial entities and to reduce spatial autocorrelation. However, by further aggregating data, researchers lose point data and the associated detail. Thus, the choice between using counties or CZs often remains subjective with insufficient empirical evidence guiding researchers in the choice. This article categorizes regional data as entrepreneurial, economic, social, demographic, or industrial and tests for the existence of local spatial autocorrelation in county and CZ data. We find CZs often reduce – but do not eliminate and can even increase – spatial autocorrelation for variables across categories. We then test the potential for regional variation in spatial autocorrelation with a series of maps and find variation based on the variable of interest. We conclude that the use of CZs does not eliminate the need to test for spatial autocorrection, but CZs may be useful for reducing spatial autocorrelation in many cases.”*
